# Validation of the EKFC equation for glomerular filtration rate estimation and comparison with the Asian-modified CKD-EPI equation in Chinese chronic kidney disease patients in an external study

**DOI:** 10.1080/0886022X.2022.2150217

**Published:** 2023-01-12

**Authors:** Li Zhao, Huan-li Li, Hui-jing Liu, Jin Ma, Wei Liu, Jian-min Huang, Ling-ge Wei, Peng Xie

**Affiliations:** a Department of Laboratory Medicine, The Third Hospital of Hebei Medical University, Shijiazhuang, PR China; b Department of ophthalmology Medicine, Hebei General Hospital, Shijiazhuang, PR China; c Department of Nuclear Medicine, The Third Hospital of Hebei Medical University, Shijiazhuang, PR China

**Keywords:** Chronic kidney disease (CKD), glomerular filtration rate (GFR), EKFC equation, Asian-modified CKD-EPI equation

## Abstract

**Objectives:**

The aim of this study is to determine whether new European Kidney Function Consortium (EKFC) equation is more applicable than Asian-modified CKD-EPI equation in clinical practice, having a higher accuracy in estimating GFR in our external CKD population.

**Methods:**

We calculated estimated GFR_EKFC_ and GFR_CKD-EPI_ independently using the EKFC and Asian-modified CKD-EPI formulas, respectively. The clinical diagnostic performance of the two equations was assessed and compared by median bias, precision, accuracy (*P*_30_) and so on, using ^99m^Tc-DTPA dual plasma sample clearance method as a reference method for GFR measurement (mGFR). The equation that met the following targets was superior: (1) median bias within ± 3 mL/min/1.73 m^2^; (2) *P*_30_ > 75%; and (3) better precision and 95% limits of agreement in Bland–Altman analysis.

**Results:**

Totally, 160 CKD patients were recruited in our external cohort. GFR_EKFC_ was highly related to mGFR, with a regression equation of GFR_EKFC_=mGFR × 0.87 + 5.27. Compared with the Asian-modified CKD-EPI equation, EKFC equation demonstrated a wider median bias (–1.64 vs. 0.84 mL/min/1.73 m^2^, *p* < 0.01) that was within 3 mL/min/1.73 m^2^ and not clinically meaningful. Furthermore, the precision (12.69 vs. 12.72 mL/min/1.73 m^2^, *p* = 0.42), 95% limits of agreement in Bland–Altman analysis (42.4 vs. 44.4 mL/min/1.73 m^2^) and incorrect reclassification index of the two target equations were almost identical. Although, EKFC equation had a slightly better *P*_30_ (80.0% vs. 74.4%, *p* = 0.01).

**Conclusions:**

The overall performance of EKFC equation is acceptable. There is no clinically meaningful difference in the performance of the Asian-modified CKD-EPI and EKFC equations within the limits imposed by the small sample size.

## Introduction

It is especially crucial for clinical laboratories to report an accurate glomerular filtration rate (GFR), which is a positive alternative for renal function assessment [1,2]. Accurate renal function assessment plays an important role in the early diagnosis, treatment adjustment, and prognostic management of chronic kidney disease (CKD) patients. GFR has been estimated by various simple creatinine-based equations in recent decades [3–5] as an alternative to inulin as the gold standard. We focus on how accurate the developed equations are in our external CKD population.

Our previous study also assessed the clinical utility of various creatinine-based equations using the ^99m^Tc-diethylenetriaminepentaacetic acid (^99m^Tc-DTPA) dual plasma sample clearance method as a reference method, which was accepted as the standard GFR assessment method at the 21st International Annual Meeting of the Society of Nuclear Medicine Europe, and demonstrated that the Asian-modified CKD-EPI equation and full age spectrum (FAS) formula were clinically more acceptable for GFR estimation with lower median bias, more superior precision and better *P*_30_ [6–9].

In 2021, Pottel et al. developed and validated a new creatinine-based equation, the new European Kidney Function Consortium (EKFC) equation [5], which can be applied to the full spectrum of age and renal function by combining the properties of the FAS [3] and CKD-EPI equations [10]. The internal development and external validation datasets suggested improved accuracy and precision compared with commonly used equations for estimating GFR from Scr levels. However, whether the EKFC equation is more accurate than the Asian-modified EPI-CKD equation for GFR estimation remains unknown, which was verified to be more accurate and convenient to use in our external CKD patients. The current study validated the EKFC equation and compared it with the Asian-modified CKD-EPI formula to recommend a more suitable equation for clinical GFR assessment in our external CKD population.

## Methods

### Ethics statement

All procedures performed in study involving human participants were in accordance with the ethical standards of the institutional and/or national research committee and with the 1964 Helsinki Declaration and its later amendments or comparable ethical standards. The study protocol was approved by the Hebei Medical University ethical committee (No. 2017-028-1), and written informed consent was obtained from each participant.

### Study subjects

This study was a prospective cohort study. Chinese patients who were diagnosed with chronic kidney disease (CKD) according to the National Kidney Foundation-Kidney Disease Outcomes Quality Initiative (K/DOQI) clinical practice guidelines [11] and who underwent ^99m^Tc-DTPA dual plasma clearance measurement were enrolled in the study cohort. We collected serum samples for the serum creatinine examination. Patients with acute kidney function deterioration, edema, cardiac insufficiency, pleural or abdominal effusion, or disabled limbs or who underwent treatment with cimetidine, trimethoprim or replacement therapy were excluded [12].

Staging criteria [13]: According to the mGFR determined by the ^99m^Tc-DTPA dual plasma sample clearance method, all patients were divided into 5 groups: normal renal function group (GFR > 90 mL/min/1.73 m^2^); mildly decreased renal function group (GFR 60–90 mL/min/1.73 m^2^); moderately decreased renal function group (GFR 30–59 mL/min/1.73 m^2^); severely decreased renal function group (GFR 15–29 mL/min/1.73 m^2^); and renal failure group (GFR <15 mL/min/1.73 m^2^).

Grouping criteria: The patients were categorized into 2 subgroups based on the mGFR calculated by the reference method: the lower-GFR subgroup (mGFR ≤ 60 mL/min/1.73 m^2^) and the higher-GFR subgroup (mGFR > 60 mL/min/1.73 m^2^).

### Laboratory measurements

#### mGFR measurement by the ^99m^Tc-DTPA dual plasma sample clearance method

At 2 and 4 h after intravenous injection of ^99m^Tc-DTPA, 3 mL heparin anti-coagulated blood samples were collected from the elbow vein, and the plasma radioactivity was measured by a multifunction well counter. Then, the clearance of ^99m^Tc-DTPA (Cl′) was calculated from a single exponential: Cl′ = [*D*×ln(*P*_1_/*P*_2_)]/(*t*_2_–*t*_1_) ×exp[(*t*_1_×ln(*P*_2_)–*t*_2_×ln(*P*_1_))/(*t*_2_–*t*_1_)]. Cl′ was corrected to GFR by Brochner–Mortensen’s formula [14]: GFR= 0.990778Cl′ − 0.001218Cl′^2^. The GFR was also standardized for a BSA of 1.73 m^2^, mGFR = GFR× (1.73/BSA), according to the Haycock formula [15] of BSA (m^2^) = 0.024265 × Wt0^.5378^ × Ht0^.3964^, using the patients’ height (cm) and weight (kg) parameters. Detailed procedures are described in a previous work [12].

#### GFR measurement by the EKFC equation

The serum creatinine (Scr) level was automatically measured by the enzymatic IDMS-traceable method using a biochemical analyzer (AU5821, Beckman, USA).

The EKFC equation is shown in Table 1 [5]. Normalized serum creatinine (Scr/*Q*) was mathematically obtained, where *Q* is the median Scr from healthy populations accounting for age and sex.

**Table 1.** EKFC equation.

Age (years)	Scr/*Q*	Equation for GFR estimation (age, years)
2–40	<1	107.3×(Scr/*Q*)^–0.322^
	≥1	107.3× (Scr/*Q*)^–1.132^
>40	<1	107.3× (Scr/*Q*)^–0.322^×0.990^(Age-40)^
	≥1	107.3× (Scr/*Q*)^–1.132^×0.990^(Age-40)^

Scr: serum creatinine concentration.

*Q* value calculations for ages 2–25 years:

Males: ln(Q) = 3.200 + 0.259 × Age − 0.543 × ln_(Age)_ − 0.00763 × Age^2^ + 0.0000790 × Age^3^.

Females: ln(Q) = 3.080 + 0.177 × Age − 0.223 × ln_(Age)_ − 0.00596 × Age^2^ + 0.0000686 × Age^3^.

*Q* value calculations for ages >25 years:

Males: *Q* = 80 µmol/L (0.90 mg/dL).

Females: *Q* = 62 µmol/L (0.70 mg/dL).

Scr and *Q* are reported in µmol/L (to convert to mg/dL, divide by 88.4).

#### GFR measurement by the Asian-modified CKD-EPI equation (GFR_CKD-EPI_)

The Asian-modified CKD-EPI equation is shown in Table 2 [4].

**Table 2.** Asian-modified CKD-EPI equation.

Gender	Scr (mg/dL)	Equation for GFR estimation (age, years)
Female	≤0.7	151× (Scr/0.7)^–0.328^× (0.993)^age^
	>0.7	151× (Scr/0.7)^–1.210^× (0.993)^age^
Male	≤0.9	149× (Scr/0.7)^–0.412^× (0.993)^age^
	>0.9	149× (Scr/0.7)^–1.210^× (0.993)^age^

Scr: serum creatinine concentration.

### Statistical analysis

Normally distributed continuous variables were described as the mean ± standard deviation (SD); otherwise, they were described as the median and interquartile range (*P*_25_–*P*_75_). Categorical variables were described as frequencies and percentages (%).

The relationship between GFR_EKFC_/GFR_CKD-EPI_ and mGFR was assessed with Spearman correlation analysis and the linear regression method. The Bland–Altman method was used to evaluate the degree of agreement between GFR_EKFC_/GFR_CKD-EPI_ and mGFR. The comparative performance indicators of GFR estimation for the EKFC equation and the Asian-modified CKD-EPI equation included median bias, precision, accuracy (*P*_30_), and incorrect reclassification index. The equation that met the following targets was considered superior: (1) median bias between ± 3 mL/min/1.73 m^2^; (2) *P*_30_ > 75%, which is considered sufficient for good clinical decision-making according to the 2002 K/DOQI benchmark [13]; and (3) better precision and 95% limits of agreement in Bland–Altman analysis. Bias and precision were defined as the difference and the interquartile range (IQR) of GFR_EKFC_/GFR_CKD-EPI_ minus mGFR, respectively. The percentage of GFR results within 30% deviation of mGFR (*P*_30_) was used as accuracy. The respective 95% confidence intervals (95% CIs) were calculated by means of bootstrap methods (2000 bootstraps) [16].

According to the patients’ basic characteristics and serum creatine values, we calculated the bias of the GFR of the EKFC and Asian-modified CKD-EPI equations using the ^99m^Tc-diethylenetriaminepentaacetic acid (^99m^Tc-DTPA) dual plasma sample clearance method as a reference method. We defined values more than 2 times the IQR of all the bias data of the two equations as outliers. We calculated the incorrect reclassification index, which was the total percentage of patients incorrectly reclassified into a different CKD stage by the EKFC equation and the Asian-modified CKD-EPI equation. The Wilcoxon signed rank test was performed to compare the median bias between the two models, the bootstrap method was used for precision comparison, and the McNemar test was used for comparison of *P*_30_ and the incorrect reclassification index. All statistical analyses were performed using IBM SPSS 21.0 (IBM Corp., Armonk, NY, USA), MATLAB software (version 2020 b, MathWorks) and the MedCalc application (version 4.3, MedCalc software, Mariekerke, Belgium). The *p* value was two-sided, and *p* < 0.05 was considered to be statistically significant.

## Results

### Characteristics of the study populations

**Figure 1.**
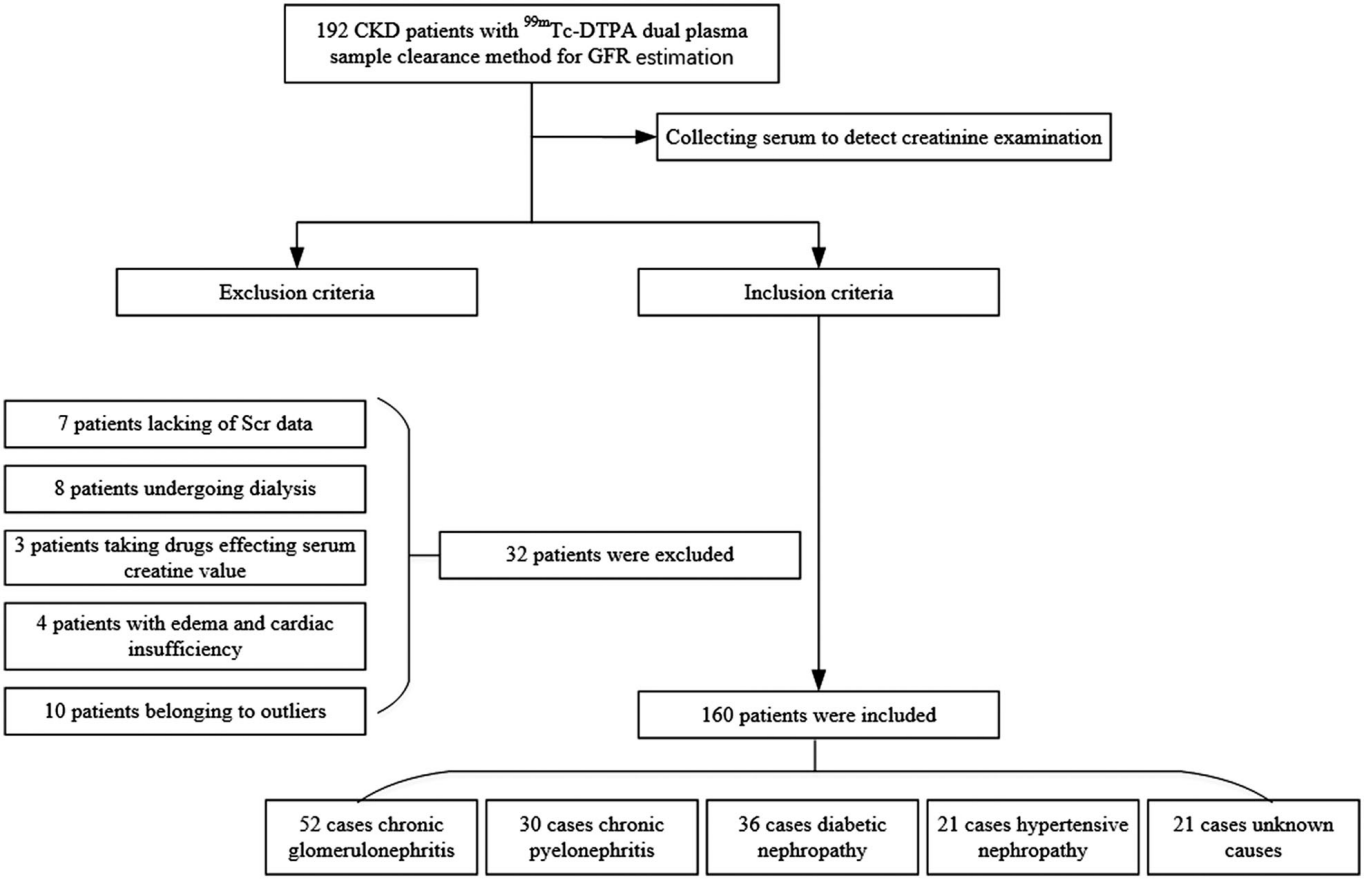
Strobe diagram of the included patients.

We collected a total of 192 CKD patients with GFR estimated by the ^99m^Tc-DTPA dual plasma sample clearance method and recollected serum samples for serum creatinine examination. However, 32 patients were excluded, 7 patients lacking Scr data due to reluctance to have their blood collected again, 8 patients undergoing dialysis, 3 patients taking drugs affecting the serum creatine value, 4 patients with edema and cardiac insufficiency, and 10 patients determined to be outliers after outlier analysis. In total, 160 patients were enrolled in our study cohort; 52 patients had chronic glomerulonephritis, 36 patients had diabetic nephropathy, 30 patients had chronic pyelonephritis, 21 patients had hypertensive nephropathy, and the remaining 21 patients had CKD due to other causes or unknown causes. The basic characteristics of the patients are shown in Figure 1 and Table 3.

**Table 3.** Basic characteristics of the study populations.

Variables	Overall group (*n* = 160)	≤60 mL/min/1.73 m^2^ subgroup (*n* = 91)	>60 mL/min/1.73 m^2^ subgroup (*n* = 69)
Male, *n* (%)	75 (46.9)	45 (49.5)	30 (43.5)
Female, *n* (%)	85 (53.1)	46 (50.5)	39 (56.5)
Age, years, *X* (SD)	56.14 (15.23)	59.98 (14.21)	51.07 (15.14)
Age range of patients (*n*)			
18 to <40 years	26	7	19
40 to 65 years	74	41	33
≥65 years	60	43	17
Height (cm), *X* (SD)	165.48 (7.96)	165.19 (7.87)	165.86 (8.13)
Weight (kg), *X* (SD)	69.00 (13.75)	68.81 (13.78)	69.25 (13.81)
Serum creatinine (mg/dL), *M* (*P*_25_–*P*_75_)	1.27 (0.91–2.32)	1.99 (1.40–3.74)	0.88 (0.78–1.03)
Male serum creatinine (mg/dL), *M* (*P*_25_–*P*_75_)	1.38 (1.10–2.68)	1.89 (1.53–3.40)	1.02 (0.91–1.21)
Female serum creatinine (mg/dL), *M* (*P*_25_–*P*_75_)	1.11 (0.80–2.27)	2.10 (1.31–3.89)	0.80 (0.72–0.90)
mGFR (mL/min/1.73 m^2^), *M* (*P*_25_–*P*_75_)	47.88 (25.37–81.27)	26.91 (16.95–42.41)	86.39 (72.28–100.28)
GFR_EKFC_ (mL/min/1.73 m^2^), *M* (*P*_25_–*P*_75_)	54.03 (24.49–78.39)	28.58 (15.28–51.30)	82.41 (65.34–94.00)
GFR_CKD-EPI_ (mL/min/1.73 m^2^), *M* (*P*_25_–*P*_75_)	56.64 (24.81–85.80)	41.78 (32.06–55.19)	88.13 (70.96–103.67)

**Table 4.** Performance comparison of the EKFC and Asian-modified CKD-EPI equations.

Indicators	Overall (mL/min/1.73 m^2^)	mGF*R* ≤ 60 mL/min/ 1.73 m^2^	mGF*R* > 60 mL/min/ 1.73 m^2^	Female (85 patients, mL/min/ 1.73 m^2^)	Male (75 patients, mL/min/ 1.73 m^2^)
Median bias, median difference (95% CI)					
Asian-modified CKD-EPI equation	0.84 (–0.63–3.60)	1.48 (–0.55–4.10)	−0.42 (–3.50–5.44)	−0.14 (–1.62–3.43)	3.14 (–0.72–6.49)
EKFC equation	–1.64 (–3.17 to −0.25), p*<0.01	−0.22 (–1.35–1.59), *p* = 0.18	−8.60 (–11.1–3.40), p*<0.01	−3.03 (–5.35–1.29), p*<0.01	−0.24 (–1.74–2.29), *p = 0.05*
Median precision, IQR of the difference (95% CI)					
Asian-modified CKD-EPI equation	12.72 (9.88–14.67)	10.25 (6.83–14.59)	15.86 (13.14–20.59)	11.26 (8.23–14.41)	15.54 (12.33–21.63)
EKFC equation	12.69 (10.38–16.61), *p* = 0.42	9.78 (6.87–12.12), p*<0.01	16.39 (12.59–19.38), p*<0.01	11.72 (7.50–15.99), p*<0.01	13.91 (9.84–18.91), p*<0.01
Accuracy, *P*_30_ (95% CI)					
Asian-modified CKD-EPI equation	74.4% (61.6–89.0%)	59.3% (44.6–77.4%)	94.2% (72.7–120.1%)	75.3% (66.1–84.5%)	73.3% (63.3–83.3%)
EKFC equation	80.0% (66.7–95.1%), p*=0.01	68.1% (52.2–87.3%), p*=0.02	95.7% (74.0–121.7%), *p* = 1.00	80.0% (71.5–88.5%), *p* = 0.13	80.0% (70.9–89.1%), *p = 0.13*

*The comparison was statistically significant between GFR_EKFC_ and GFR_CKD-EPI_.

### Clinical assessment of the EKFC equation

**Figure 2.**
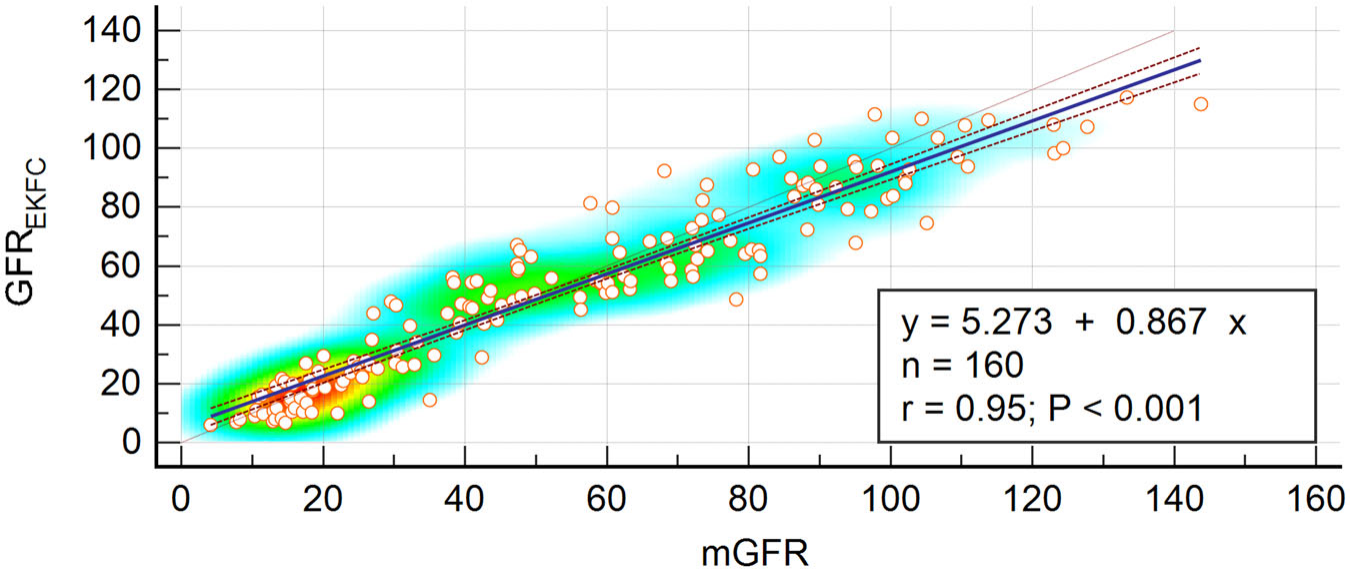
Scatter plots and regression equation of GFR_EKFC_ and mGFR (mL/min/1.73 m^2^). mGFR is located on the *X* axis, and GFR_EKFC_ is located on the *Y* axis. The solid blue line represents the regression line between GFR_EKFC_ and mGFR, and dashed red lines represent 95% confidence intervals for the regression line. The solid red line represents the identity line of *y* = *x.*

**Figure 3.**
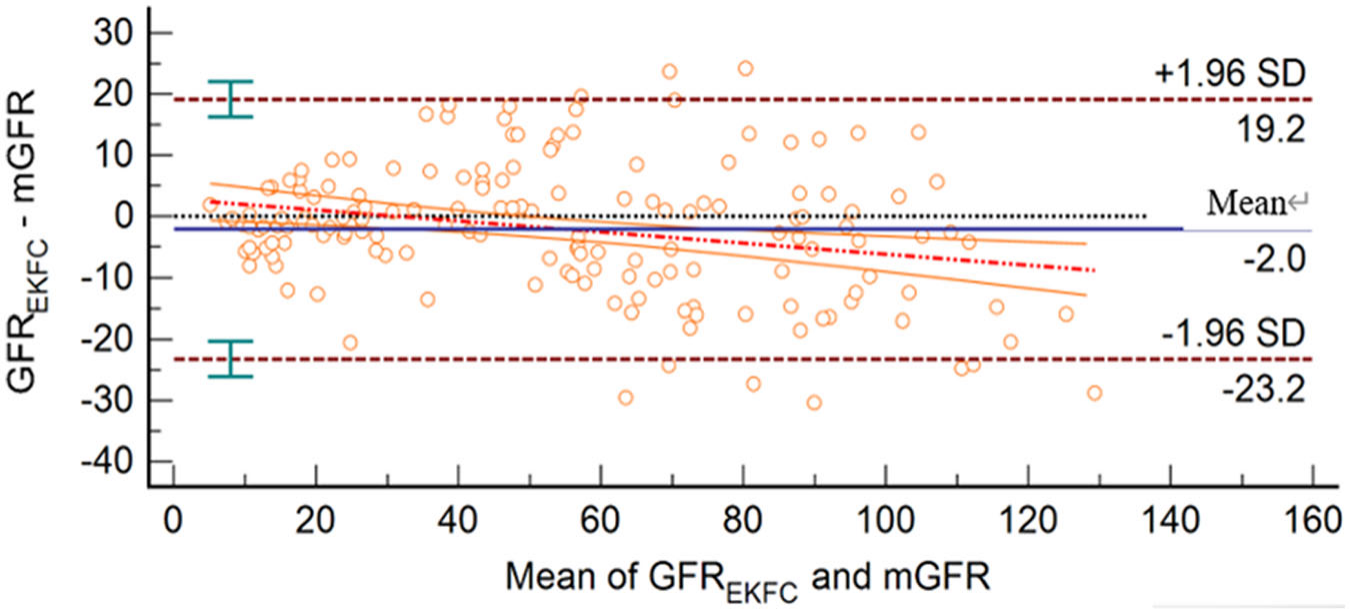
Bland–Altman plot of GFR_EKFC_ and mGFR (mL/min/1.73 m^2^). The mean of mGFR plus GFR_EKFC_ is located on the *X* axis, and the value of mGFR minus GFR_EKFC_ is located on the *Y* axis. The solid blue line represents the mean difference between methods, the dashed dark red lines represent 95% limits of agreement of the mean difference between methods, the dotted red line represents the regression line of the difference between methods against mGFR, and the solid orange lines represent 95% confidence intervals for the regression line.

GFR_EKFC_ was highly related to mGFR, with a correlation coefficient of 0.95 (95% CI, 0.93–0.96) and a regression equation of GFR_EKFC_=mGFR × 0.87 + 5.27 (Figure 2). The Bland–Altman plot showed that the 95% limits of agreement for the EKFC equation were −23.2 to 19.2 mL/min/1.73 m^2^ (Figure 3).

### Performance comparison of the EKFC equation and the Asian-modified CKD-EPI equation for GFR estimation

**Figure 4.**
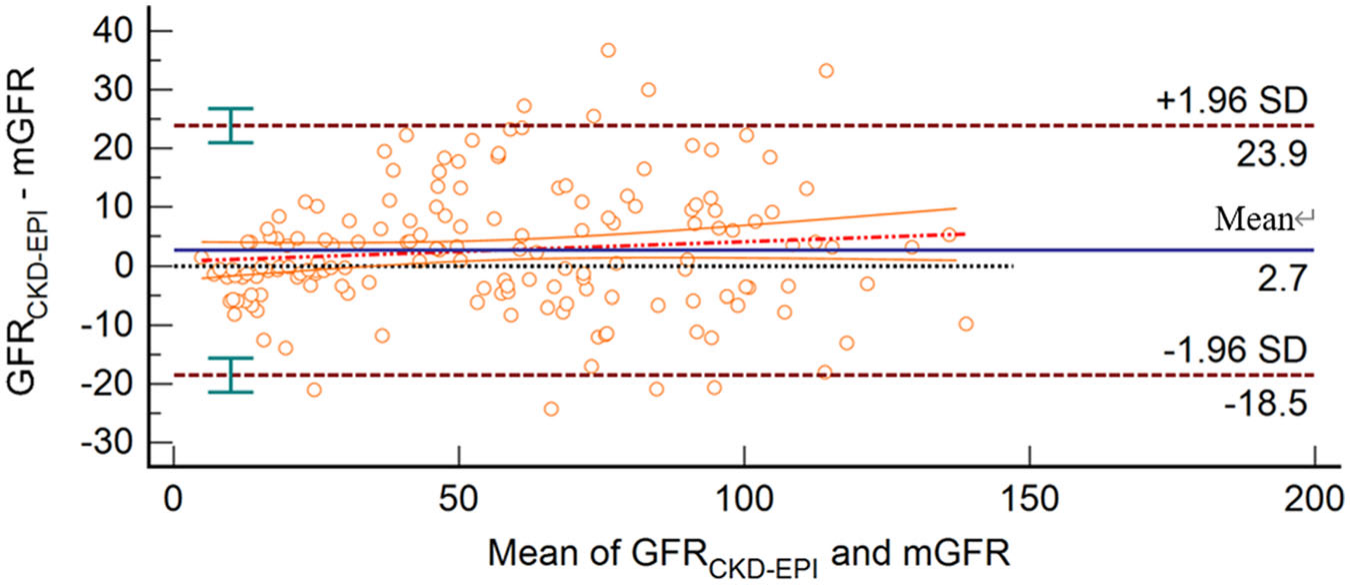
Bland–Altman plot of GFR_CKD-EPI_ and mGFR (mL/min/1.73 m^2^). The mean mGFR plus GFR_CKD-EPI_ is located on the *X* axis, and the value of mGFR minus GFR_CKD-EPI_ is located on the *Y* axis. The solid blue line represents the mean difference between methods, the dashed dark red lines represent 95% limits of agreement of the mean difference between methods, the dotted red line represents the regression line of the difference between methods against mGFR, and the solid orange lines represent 95% confidence intervals for the regression line.

The linear relationship between GFR_CKD-EPI_ and mGFR was GFR_CKD-EPI_=0.98 × mGFR +3.75. The Bland–Altman plot demonstrated that the 95% limits of agreement for the Asian-modified CKD-EPI equation were −18.5 to 23.9 mL/min/1.73 m^2^ (Figure 4). The 95% limits of agreement in Bland–Altman analysis of the EKFC and Asian-modified CKD-EPI equations were almost the same (42.4 vs. 44.4 mL/min/1.73 m^2^). However, compared with the EKFC equation, the slope of the regression equation of the Asian-modified CKD-EPI equation was closer to 1, and the intercept approached zero.

Compared with the Asian-modified CKD-EPI equation, the EKFC equation demonstrated a wider median bias (–1.64 vs. 0.84 mL/min/1.73 m^2^, *p* < 0.01), which was still within 3 mL/min/1.73 m^2^ and was clinically not meaningful, although it was statistically significant. Furthermore, precision (12.69 vs. 12.72 mL/min/1.73 m^2^, *p* = 0.42) and the incorrect reclassification index in the different stages of CKD between the two models were not significantly different. However, the EKFC equation had a slightly better *P*_30_ (80.0% vs. 74.4%, *p* = 0.01).

In the mGFR ≤ 60 mL/min/1.73 m^2^ subgroup analysis, the precision (9.78 vs. 10.25 mL/min/1.73 m^2^, *p* < 0.01) and *P*_30_ (68.1% vs. 59.3%, *p* = 0.02) of the EKFC equation were better than those of the Asian-modified CKD-EPI equation, but the median bias was not significantly different (1.48 vs. −0.22 mL/min/1.73 m^2^, *p* = 0.18). However, in the mGFR > 60 mL/min/1.73 m^2^ subgroup, the EKFC equation did not perform better than the Asian-modified CKD-EPI equation, with a wider median bias (–8.60 vs. −0.42 mL/min/1.73 m^2^, *p* < 0.01), an inferior precision (16.39 vs. 15.86 mL/min/1.73 m^2^, *p* < 0.01), and a statistically nonsignificant *P*_30_ (95.7% vs. 94.2%, *p* = 1.00).

Furthermore, we compared the performance of the two equations in males and females and found that the results of the two equations were not different, with a nonmeaningful median bias and insignificantly different P_30._ However, the precision of the Asian-modified CKD-EPI equation was better in females than males, and the EKFC equation was superior in males. The performance comparison between the EKFC equation and the Asian-modified CKD-EPI equation is summarized in Tables 4 and 5.

**Table 5.** Incorrect reclassification results between the two models.

CKD stage (mL/min/1.73 m^2^)	Patients, *n*	mGFR (mL/min/1.73 m^2^)	GFR_EKFC_, *M* (*P*_25_–*P*_75_) (mL/min/1.73 m^2^)	GFR_CKD-EPI_, *M* (*P*_25_–*P*_75_) (mL/min/1.73 m^2^)	EKFC equation Incorrectly reclassified, n (%)	Asian-modified CKD-EPI equation Incorrectly reclassified, n (%)	*p* value
<15	17	12.89 (10.42–13.80)	9.86 (7.61–15.85)	9.92 (7.22–15.20)	5 (29.4%)	4 (23.5%)	0.74
15–29	32	21.13 (17.62–25.55)	21.13 (14.15–26.05)	21.55 (13.98–27.02)	11 (34.4%)	12 (37.5%)	0.83
30–59	42	42.98 (38.03–48.28)	48.68 (40.16–55.05)	51.56 (43.50–60.69)	11 (26.2%)	15 (35.7%)	0.44
60–90	41	73.54 (68.27–81.65)	68.44 (58.82–83.05)	74.57 (65.22–89.93)	11 (26.8%)	14 (34.1%)	0.56
>90	28	102.37 (97.37–113.05)	94.93 (87.25–107.72)	105.36 (96.23–116.31)	5 (17.9%)	5 (17.9%)	1.00

## Discussion

The Kidney Disease Outcomes Quality Initiative (K/DOQI) guidelines from 2002 clearly indicated that the patient’s GFR was the primary determinant of the diagnostic rationale and staging criteria for CKD patients. Several formulas in current clinical practice were constructed to estimate GFR. In 2021, Pottel et al. developed and validated the EKFC equation, which was a modified full age spectrum, Scr-based GFR estimation equation. How accurate the EKFC equation is in external populations was the main purpose of our study.

In the current study, we assessed the clinical utility of the EKFC equation and demonstrated that it was acceptable in clinical practice. GFR_EKFC_ was highly related to mGFR; the regression coefficient was near 1, and the intercept was close to zero. Furthermore, the Bland–Altman plot showed that the 95% limits of agreement for the EKFC equation were satisfactory in our external population. As a full age spectrum equation, the new EKFC equation provides continuity across the entire age range and facilitates GFR reporting in the clinical laboratory by simple calculation. Compared with the Asian-modified CKD-EPI equation, the EKFC equation demonstrated a wider median bias within the range of ±3 mL/min/1.73 m^2^, and the difference had no clinical value. Despite a slightly better *P*_30_ of 80%, the EKFC equation demonstrated nearly identical precision and was not significantly different in the incorrect reclassification index compared with the Asian-modified CKD-EPI equation. In total, there is no clinically meaningful difference in the performance of the two equations in our external Chinese chronic kidney disease population. In the mGFR ≤ 60 mL/min/1.73 m^2^ subgroup, the EKFC equation produced excellent results. However, its performance was relatively disappointing in the mGFR > 60 mL/min/1.73 m^2^ subgroup, with a wider median bias and inferior precision compared with the Asian-modified CKD-EPI formula in our external CKD patients.

Furthermore, we compared the performance of the two equations in males and females and found that there were no differences in sex. The reason for this could be that the EKFC equation used Q values that were independently different for males and females, and the Asian-modified equation was developed for females and males separately.

Overall, the EKFC equation was clinically acceptable in our external Chinese CKD patient cohort. The overall bias of −1.64 mL/min/1.73 m^2^ was not different from the bias of −0.9 mL/min/1.73 m^2^ that was reported in the external validation dataset in the original study. However, the *P*_30_ of 80% in our study was lower than the overall 88% in the original study. The reason why the *P*_30_ of the EKFC equation was worse in our external population may be because the EKFC equation was developed based on a cross-sectional analysis of pooled data, from which many algorithms were developed by different reference methods, for example, ^99m^Tc-DTPA renal dynamic imaging. In fact, a large number of studies have shown that ^99m^Tc-DTPA renal dynamic imaging for GFR is not ideal and should not be used as a reference standard [17] because it may introduce errors in the process of developing and assessing the EKFC equation. In our validation cohort, we used the ^99m^Tc-DTPA dual plasma clearance method as the reference standard, which was accepted as the standard GFR method at the 21st International Annual Meeting of the Society of Nuclear Medicine Europe. The utilization of a different gold standard may have caused the different validation results in our external cohort. Additionally, in the mGFR > 60 mL/min/1.73 m^2^ subgroup, the EKFC equation had a wider median bias than those of the Asian-modified CKD-EPI equation and the original external validation dataset. We found that the median bias of the EKFC equation was higher in the mGFR > 60 mL/min/1.73 m^2^ subgroup than in the mGFR ≤ 60 mL/min/1.73 m^2^ subgroup. It is possible that the higher the mGFR is, the more obvious the underestimation of the EKFC equation, as shown in Figure 2. Furthermore, the EKFC equation was developed and validated in white populations. Our study population included participants of Asian ethnicity; the median Scr concentration is different among the various ethnicities, which leads to a different *Q* value, where *Q* represents the median creatine value of healthy persons, and thus distinct GFR according to the EKFC equation. In future work, we will calculate the *Q* value according to the Asian median Scr concentration and further validate the EKFC equation with a larger sample size.

Our study has some limitations. First, our population’s sample size was not sufficient. Second, our study included Asian adult CKD patients; therefore, we could not determine the accuracy of the EKFC equation in children’s populations. More external validating studies are needed to verify the clinical application of the EKFC equation.

## Conclusions

Overall, for adult CKD patients, there is no clinically meaningful difference in the performance of the two equations within the limits imposed by the small sample size. Clinicians should assess the GFR of adult CKD patients based on the patient’s actual situation in combination with the equation in clinical practice. For adolescents and children with CKD, more external validation studies are needed to assess the accuracy of the EKFC equation.

## Data Availability

The datasets used and/or analyzed during the current study are available from the correspondence author on reasonable request.
